# Supplementary material for: DEEPMIR: a deep neural network for differential detection of cerebral microbleeds and iron deposits in MRI
Source: Sci Rep. 2021 Jul 8;11:14124. doi: 10.1038/s41598-021-93427-x (PMC8266884; doi:10.1038/s41598-021-93427-x)
Supplement: Supplementary file 1 — Supplementary Information. [file 41598_2021_93427_MOESM1_ESM.docx]

# **Supplementary Materials**

**Title:**

DEEPMIR: A Deep Neural Network for Differential Detection of Cerebral Microbleeds and IRon Deposits in MRI

**Authors:**

Tanweer Rashid^1,2^*, Ahmed Abdulkadir^2,3^, Ilya M. Nasrallah^2,4^, Jeffrey B. Ware^4^, Hangfan Liu^2^, Pascal Spincemaille^5^, J. Rafael Romero^6^, R. Nick Bryan^4,7^, Susan R. Heckbert^8^, Mohamad Habes^1,2^*

**Affiliations:**

^1^Neuroimage Analytics Laboratory (NAL) and the Biggs Institute Neuroimaging Core (BINC), Glenn Biggs Institute for neurodegenerative disorders, University of Texas Health Science Center at San Antonio (UTHSCSA), San Antonio, Texas, USA

^2^Center for Biomedical Image Computing and Analytics (CBICA), University of Pennsylvania, Philadelphia, PA, USA

^3^University Hospital of Old Age Psychiatry and Psychotherapy, University of Bern, Bern, Switzerland

^4^Department of Radiology, Hospital of University of Pennsylvania, Perelman School of Medicine of the University of Pennsylvania, Philadelphia, PA, USA

^5^Department of Radiology, Weill Cornell Medical College, New York, NY, USA

^6^Department of Neurology, School of Medicine, Boston University, Boston, MA, USA

^7^Department of Diagnostic Medicine, Dell Medical School, University of Texas at Austin, Austin, TX, USA

^8^Department of Epidemiology and Cardiovascular Health Research Unit, University of Washington, Seattle, WA, USA

**Corresponding authors: habes@uthscsa.edu and rashidt1@uthscsa.edu**

# Section 1. Multi-Echo SWI

Susceptibility weighted imaging (SWI) is a high-resolution, 3D imaging technique where the magnitude image is combined with phase data (in the post-processing stage), and is able to detect small CMBs due to its’ high sensitivity to haemosiderin^1^. The choice of the echo time has a significant impact on the visibility and/or detection of CMBs. Longer echo times allow increased proton dephasing, (also known as the blooming effect) and may increase the CMB’s hypo-intensity and/or size. However, there are disadvantages in the use of longer TEs. Blood vessels that are perpendicular to the imaging plane may take on the appearance of CMBs (i.e. hypo-intense and rounded in shape), and for longer TE even small blood vessels may be visible and thus increase the chances of false positive identification in an automated segmentation/detection algorithm. Supplementary Figure 1 shows an example of a CMB mimic becoming more visible at longer TEs. Another side effect of the use of longer TEs is diminishing image quality. The presence of air-tissue interfaces in regions such as the sinus cavity and skull base can cause distortions/artifacts in the image and create uncertainty in distinguishing CMBs from noise. Supplementary Figure 2 shows an example of increasing distortions in the region above the sinus cavity for longer TE, and Supplementary Figure 3 shows an example of poor image quality for regions in the temporal lobe and cerebellum at longer TEs. These distortions or residual phase wrap errors are likely caused by the choice of homodyne high-pass filtering and small filter size of 64x64^2,3^.


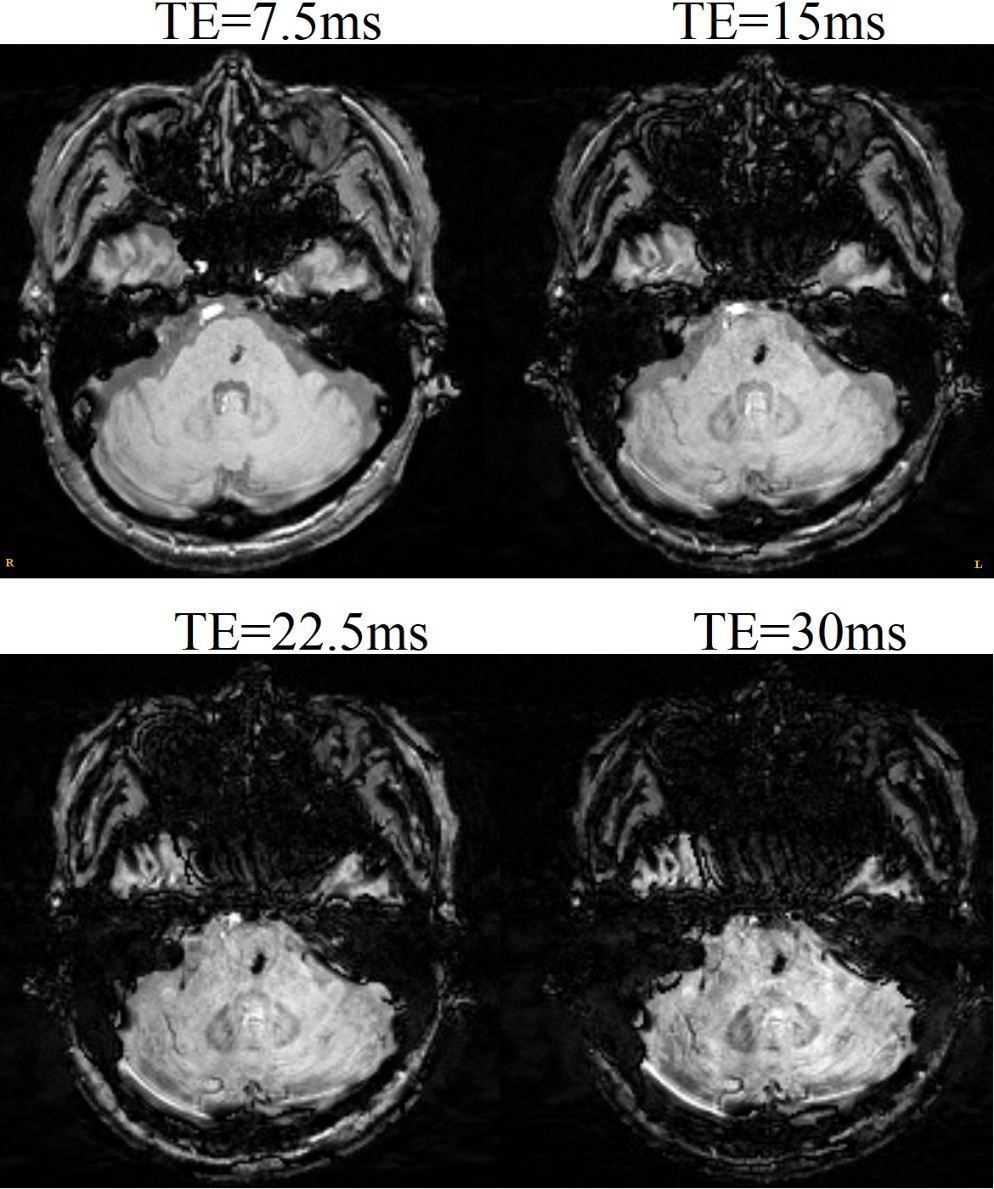


Supplementary Figure 1: Example of a blood vessel having more pronounced visibility in SWI with longer TEs. There is a true CMB (red arrow) and a potential CMB mimic (yellow arrow) which is not visible at short TEs but becomes more visible at longer TEs.


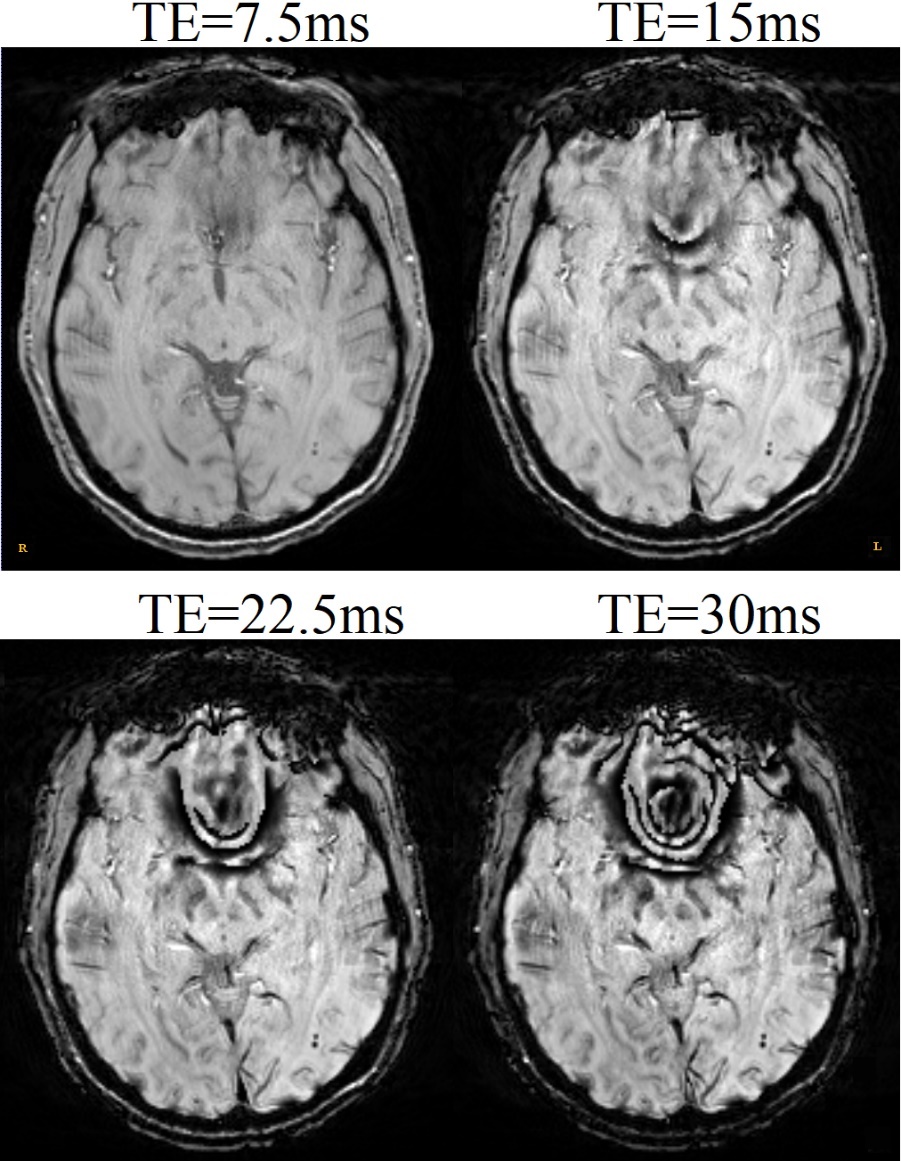


Supplementary Figure 2: Example of distortions (residual phase wrap errors) in the region above the sinus cavity for SWIs with longer TEs.


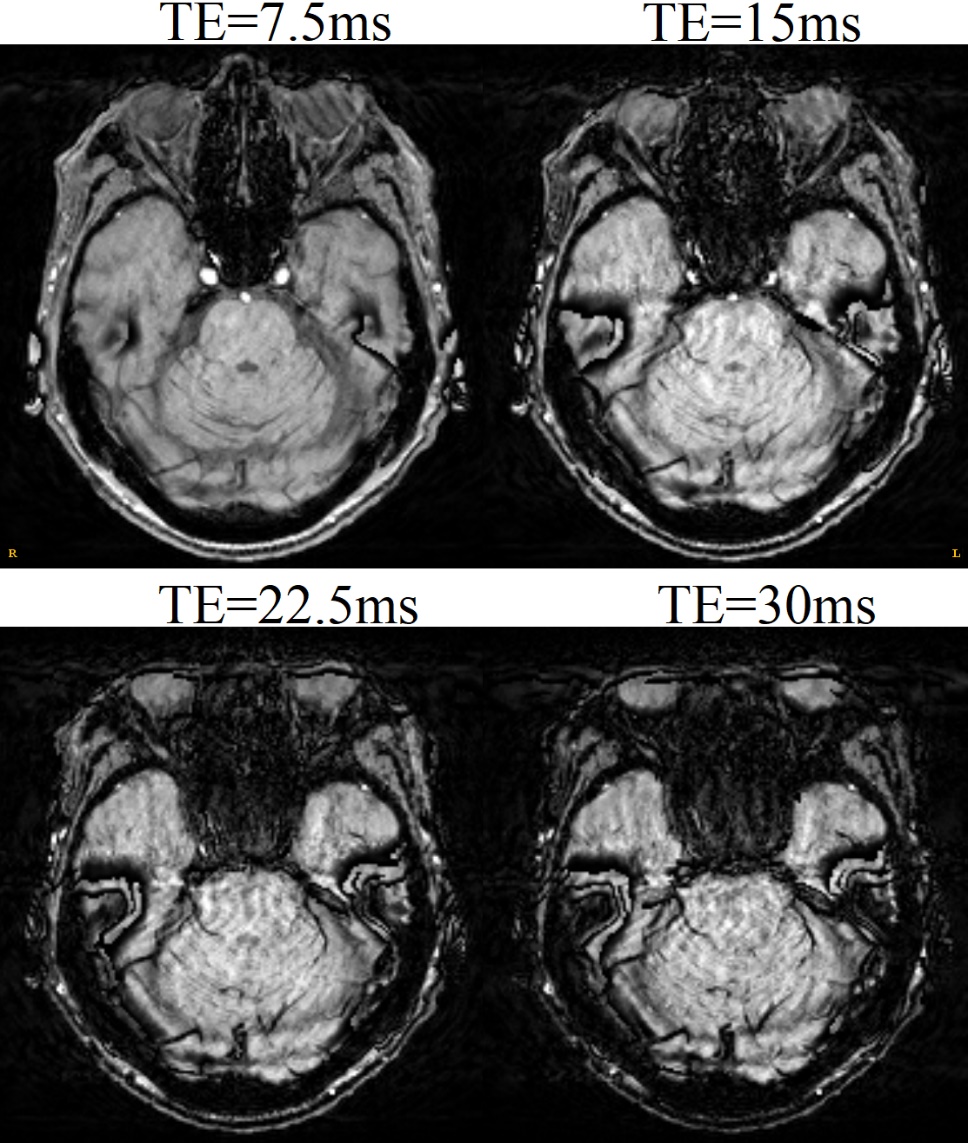


Supplementary Figure 3: An example of increasing distortions (residual phase wrap errors) in the temporal lobes and cerebellum for SWIs with longer TEs.

Supplementary Table 1.1: Scanner parameters.

| **Image Type** | **Echo Time (TE) (ms)** | **Repetition Time (TR) (ms)** | **Pixel Bandwidth (Hz/pixel)** | **Flip Angle (FA)** | **Slice Thickness (mm)** | **Acquisition Matrix** | **in-plane voxel size (mm)** | **Number of Slices** | **Acquisition Type** |
| --- | --- | --- | --- | --- | --- | --- | --- | --- | --- |
| **T1w MPRAGE** | 2.93 | 1900 | 170 | 9 | 1 | 256x256 | 1x1 | 176 | 3D |
| **T2w** | 408 | 3200 | 750 | 120 | 1 | 256x256 | 1x1 | 176 | 3D |
| **SWI** | 7.5, 15, 22.5, and 30 | 35 | 200 | 15 | 1.5 | 256x192 | 1x1 | 96 | 3D |

# Section 2. Manual Annotation

## 2.1 Manual Annotation of CMBs and Non-hemorrhage Iron Deposits

Current rating scales for CMBs such as MARS^4^ and BOMBS^5^ provided only coarse information about the spatial location and shape of CMBs. They were not designed to individually detect and annotate individual CMBs on the MR image itself, which limits their usefulness for training machine learning models. To address this, we implemented a protocol (shown in Supplementary Figure 4) to simultaneously annotate CMBs and non-hemorrhage iron deposits in the basal ganglia using SWI, QSM and T2w MRI. Our protocol was inspired by the systemic approach of the Brain Observer MicroBleed Scale (BOMBS)^5^. CMBs and non-hemorrhage iron deposits were annotated by experts (IMN and TR) with integer values 1 and 2, respectively. The manual annotation is based on the following observations:

1. CMBs and iron deposits are hypo-intense on SWI, hyper-intense on QSM and have some hypo-intensity on T2w,
2. CMBs are round in shape and can appear anywhere in the brain,
3. Non-hemorrhage iron deposits do not have any specific shape but are generally larger than CMBs and are mainly located in the gray matter of the basal ganglia, particularly the globus pallidus
4. Similar to the characteristics of CMBs and iron deposits described in MARS, if a hypo-intensity on SWI occurs unilaterally, i.e. on one side of the basal ganglia, then it is more likely to be a CMB, and if the hypo-intensity is bilateral, then it is assumed to be non-hemorrhage iron deposits.

The annotation protocol is as follows: For each axial SWI slice having a round hypo-intense region (candidate region) similar to CMBs:

1. Inspect the previous and next few slices to ensure that the round hypo-intense region is not a part of other structures such as blood vessels or sulcus. If it is a part of a blood vessel or similar elongated linear structure, then the rounded hypointense region will be prevalent in several adjacent axial slices (more than 5 slices). If the rounded hypointense region is part of the sulci, then the hypointense region will seem to join with the sulci in subsequent slices. It may be necessary to inspect the candidate region in sagittal and coronal slices to verify.
2. Once it has been confirmed that the round hypo-intense region is separate and not part of other structures, check the region’s corresponding intensities on the T2w and QSM images.
   1. If the corresponding region is hyper-intense on the T2w image, and shows no appreciable change in intensity on the QSM compared to surrounding voxels, then the region likely represents an enlarged perivascular space.
   2. If the corresponding region is hypo-intense or does not show any discernable changes in intensity on the T2w compared to surrounding voxels, and hyperintense on the QSM image, then the region likely represents a CMB.
3. If the region within the globus pallidus section of the basal ganglia is hypo-intense on the SWI, hyper-intense on the QSM, and show some hypo-intensity on the T2w image compared to surrounding voxels, then the region represents non-hemorrhage iron deposits. On the other hand, if the region is hypo-intense on the QSM, then the region likely represents calcium deposits.

Within

Basal Ganglia?

Intensity on T2w?

Intensity on QSM?

Has rounded shape?

(check neighboring slices)

Hypo-intense region

separate from neighboring structures?

Separable from Sulcus?

(Check **sagittal** and **coronal** slices)

Intensity on T2w?

Intensity on QSM?

Start

Next axial **SWI** slice

Label region as **Cerebral Microbleed**

Label region as

**Iron Deposit**

**Enlarged Perivascular Space**

**(Do nothing)**

Yes

No

Found **hypo-intense** voxels/region

in slice?

Hypo-intense

Yes

Hyper-intense

Hyper-intense

Yes

Hypo-intense

Yes

No

No

Hyper-intense

Hyper-intense

**Basal Ganglia Calcification**

**(Do nothing)**

No

Hypo-intense

Hypo-intense

Hypo-intensity unilateral or bilateral?

Bilateral

Unilateral

Supplementary Figure 4: Flowchart for labeling cerebral microbleeds, iron deposits, basal ganglia calcifications and enlarged perivascular spaces.

## 2.2 Participant Information

Supplementary Table 2.1: Summary demographics for the included MESA participants (n=24).

| **Participants** | **Age** | **Sex** | **Number of CMBs**  **(Average Size)** | **Iron deposits (voxel count)** |
| --- | --- | --- | --- | --- |
| 4 participants | 74-89 | 3 females, 1 male | 0 CMBs  (0 voxels or 0 mm^3^) | 4 participants had 96 – 326 voxels |
| 13 participants | 66-94 | 6 females, 7 males | 1 or 2 CMBs  (7.24 voxels or 10.85 mm^3^) | 11 participants had 9 – 283 voxels, 2 participants had 0 voxel) |
| 6 participants | 65-89 | 2 females, 4 males | 3 to 8 CMBs  (7.1 voxels or 10.21 mm^3^) | 4 participants had 2 – 146 voxels, 2 participants had 0 voxels |
| 1 participant | 67 | 1 male | 120  (3.175 voxels or 4.76 mm^3^) | 0 voxels |


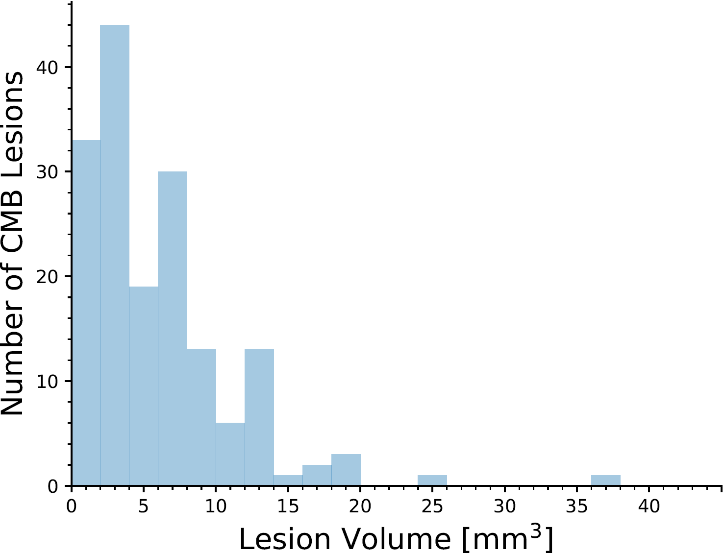

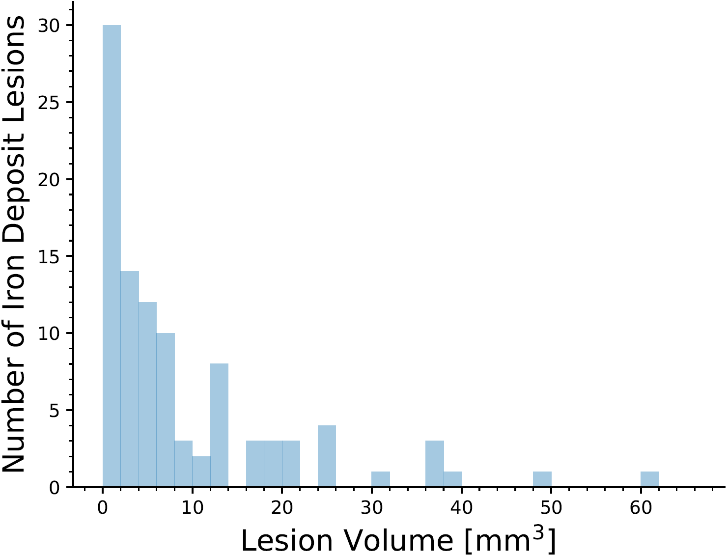


Supplementary Figure 5:Histogram of the size of cerebral microbleeds (left panel) and iron deposits (right panel) lesions in mm^3^ pooled over all participants.

# Section 3. Experimental Results Excluding Outlier

## 3.1 Outlier Detection

In our training and testing dataset, there is a single participant with more than 100 CMBs. As shown in Supplementary Figure 6, a beta distribution fitted to the number of CMBs in this dataset indicates that the 99^th^ percentile of the distribution is approximately 16 CMBs. The participant with more than 100 CMBs is clearly an outlier by this definition.

It should be noted that under different circumstances this participant may not be considered an outlier in terms of the number of CMBs. Large numbers of CMBs have been observed in patients with vascular pathologies such as cerebral amyloid angiopathy (CAA)^6^ or hypertension^7^.

## 3.2 Experiments Results

In this series of experiments, model training and testing using leave-one-out cross-validation was conducted with the outlier participant being excluded from the dataset. All other training and testing parameters were kept the same as the experiments in the main paper. The non-parametric two-tailed Wilcoxon signed rank test^8^ was used to check for statistical significance in the average sensitivity, precision and magnitude accuracy in all experiments. Comparisons were made against the model trained with only SWI. All statistical testing was performed using MATLAB R2017b.

The results of all single class experiments are reported in Supplementary Table 3.1. For these experiments, we note that model trained with SWI, QSM and T2w had the best performance in terms of average magnitude accuracy for detecting CMBs. For detecting iron deposits, the model trained with SWI and QSM had the highest average magnitude accuracy. Supplementary Figure 7 shows the scatterplots of the average sensitivity and precision for all single class experiments.

The results of all multiclass experiments are reported in Table 3.2. Interestingly, the model trained with SWI and T2w had the best performance in terms of average magnitude accuracy for both CMBs and iron deposits. Supplementary Figure 8 shows the scatterplots of the average sensitivity and precision for all multiclass experiments.


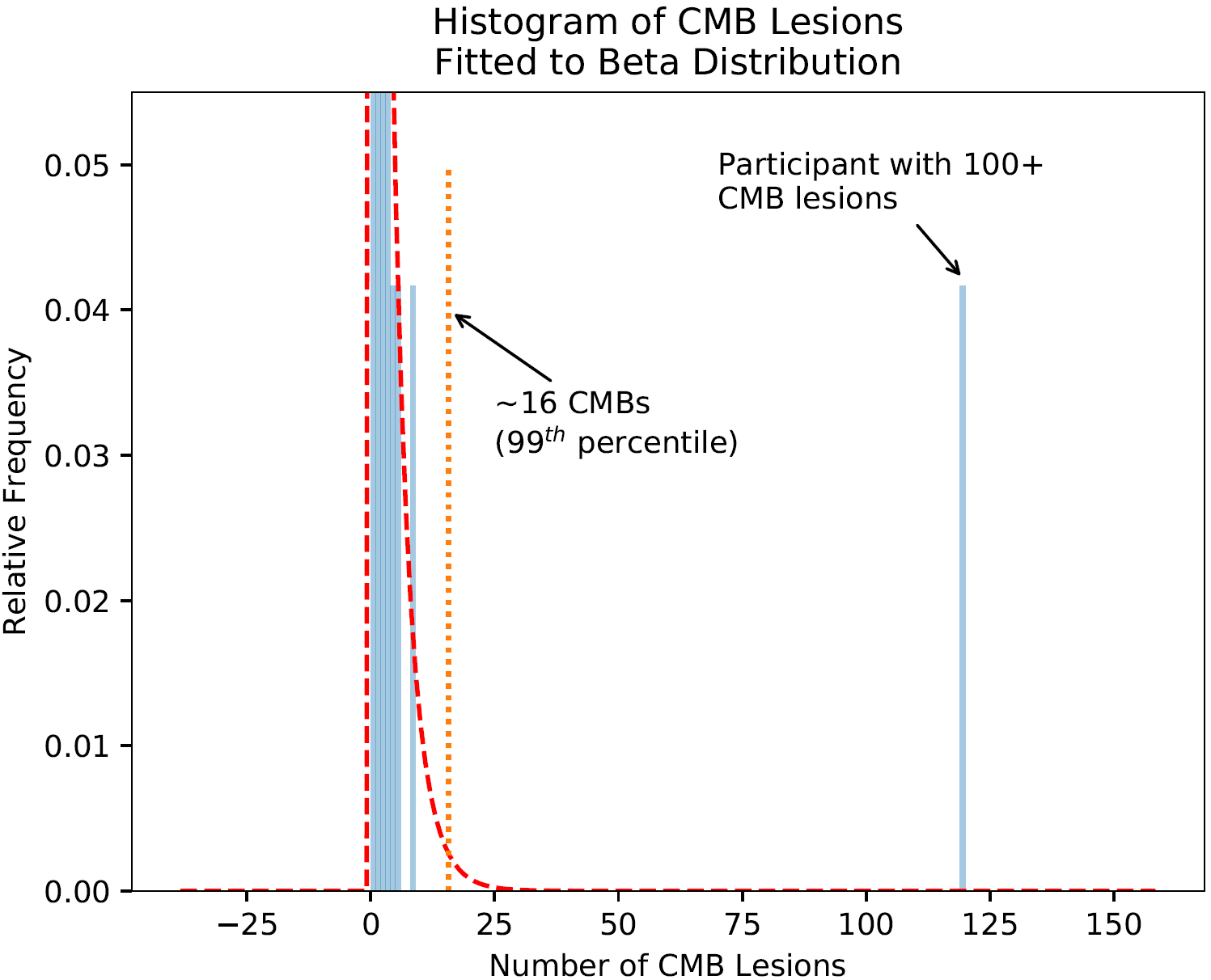


Supplementary Figure 6: A histogram of the number of CMB lesions. The blue columns represent the number of lesions. A beta distribution is fitted to this histogram (red dashed curve). The 99^th^ percentile of this distribution (orange dotted line) is approximately 16 CMBs. The blue bar towards the right of the figure is the participant with more than 100 CMBs.

Supplementary Table 3.1: Experimental result using the single class model for the number of predicted CMB and iron deposit lesions evaluated against the reference annotation when the outlier is excluded.

|  | **Experiments** | **Avg Sensitivity ± SEM [CI: lower, upper]** | **Avg Precision ± SEM [CI: lower, upper]** | **Avg Magnitude Accuracy ± SEM [CI: lower, upper]** | **Pearson**  **Correlation Coeff**  **(p-value)** | **Bland-Altman Plot (md, [lower, upper])** |
| --- | --- | --- | --- | --- | --- | --- |
| **Single Class CMB** | SWI | 0.76 ± 0.09  [0.59, 0.93] | 0.30 ± 0.06  [0.18, 0.43] | 0.86 ± 0.10  [0.67, 1.05] | 0.27  (p=0.219) | md=-4.09  CI: [-15.22, 7.04] |
|  | SWI and QSM | 0.81 ± 0.08  [0.65, 0.96] | 0.43 ± 0.07  [0.29, 0.57] | 0.97 ± 0.09  [0.79, 1.14] | 0.51  (p=0.012) | md=-1.52  CI: [-5.88, 2.84] |
|  | SWI and T2w | 0.86 ± 0.07  [0.72, 1.00] | 0.35 ± 0.07  [0.22, 0.49] | 0.98 ± 0.09  [0.81, 1.15] | 0.27  (p=0.220) | md=-4.04  CI: [-14.69, 6.61] |
|  | **SWI, QSM and T2w** | **0.85 ± 0.07**  **[0.71, 0.99]** | **0.45 ± 0.08**  **[0.29, 0.60]** | **1.09 ± 0.07**  **[0.94, 1.23]** | **0.47**  (p=0.022) | **md=-2.74**  **CI: [-9.58, 4.10]** |
|  |  |  |  |  |  |  |
| **Single Class Iron Deposits** | SWI | 0.80 ± 0.06  [0.68, 0.93] | 0.54 ± 0.07  [0.40, 0.68] | 1.05 ± 0.08  [0.90, 1.20] | 0.92  (p=0.000) | md=0.52  CI: [-77.23, 78.27] |
|  | **SWI and QSM** | **0.80 ± 0.06**  **[0.68, 0.92]** | **0.64 ± 0.07**  **[0.50, 0.77]** | **1.12 ± 0.05**  **[1.01, 1.22]** | **0.94**  (p=0.000) | **md=1.35**  **CI: [-64.33, 67.02]** |
|  | SWI and T2w | 0.74 ± 0.07  [0.60, 0.89] | 0.51 ± 0.07  [0.37, 0.65] | 0.99 ± 0.08  [0.83, 1.16] | 0.87  (p=0.000) | md=3.13  CI: [-88.11, 94.37] |
|  | SWI, QSM and T2w | 0.76 ± 0.07  [0.62, 0.90] | 0.56 ± 0.08  [0.41, 0.71] | 1.06 ± 0.07  [0.92, 1.20] | 0.85  (p=0.000) | md=-3.35  CI: [-111.66, 104.96] |

SEM = standard error of the mean

md = mean difference

CI = confidence interval

**Bold – Model with highest magnitude accuracy**

Supplementary Table 3.2: Experimental result using the multiclass model for the number of predicted CMB and iron deposit lesions evaluated against the reference annotation when the outlier is excluded.

|  | **Experiments** | **Avg Sensitivity ± SEM [CI: lower, upper]** | **Avg Precision ± SEM [CI: lower, upper]** | **Avg Magnitude Accuracy ± SEM [CI: lower, upper]** | **Pearson**  **Correlation Coeff**  **(p-value)** | **Bland-Altman Plot (md, [lower, upper])** |
| --- | --- | --- | --- | --- | --- | --- |
| **Multiclass CMB** | SWI | 0.72 ± 0.08  CI: [0.56, 0.88] | 0.46 ± 0.06  CI: [0.34, 0.59] | 1.03 ± 0.04  CI: [0.96, 1.11] | 0.56  (p=0.005) | md=-0.96  CI: [-4.90, 2.98] |
|  | SWI and QSM | 0.85 ± 0.07  CI: [0.71, 0.98] | 0.55 ± 0.08  CI: [0.39, 0.71] | 1.11 ± 0.08  CI: [0.96, 1.26] | 0.69  (p=0.000) | md=-1.26  CI: [-4.97, 2.45] |
|  | **SWI and T2w** | **0.86 ± 0.06**  **CI: [0.74, 0.98]** | **0.62 ± 0.07**  **CI: [0.48, 0.77]** | **1.18 ± 0.04**  **CI: [1.10, 1.27]** | **0.69**  (p=0.000) | **md=-0.83**  **CI: [-3.97, 2.32]** |
|  | SWI, QSM and T2w | 0.89 ± 0.06  CI: [0.78, 1.00] | 0.48 ± 0.07  CI: [0.34, 0.61] | 1.07 ± 0.07  CI: [0.93, 1.21] | 0.60  (p=0.002) | md=-1.96  CI: [-7.66, 3.75] |
|  |  |  |  |  |  |  |
| **Multiclass Iron Deposits** | SWI | 0.84 ± 0.05  CI: [0.74, 0.93] | 0.65 ± 0.07  CI: [0.51, 0.79] | 1.16 ± 0.05  CI: [1.07, 1.26] | 0.95  (p=0.000) | md=14.52  CI: [-52.73, 81.77] |
|  | SWI and QSM | 0.80 ± 0.07  CI: [0.67, 0.94] | 0.66 ± 0.07  CI: [0.52, 0.80] | 1.17 ± 0.05  CI: [1.07, 1.27] | 0.97  (p=0.000) | md=6.70  CI: [-45.57, 58.96] |
|  | **SWI and T2w** | **0.87 ± 0.04**  **CI: [0.78, 0.95]** | **0.66 ± 0.07**  **CI: [0.52, 0.79]** | **1.17 ± 0.05**  **CI: [1.08, 1.26]** | **0.93**  (p=0.000) | **md=18.78**  **CI: [-57.96, 95.53]** |
|  | SWI, QSM and T2w | 0.77 ± 0.08  CI: [0.62, 0.93] | 0.56 ± 0.07  CI: [0.42, 0.71] | 1.06 ± 0.09  CI: [0.89, 1.23] | 0.91  (p=0.000) | md=13.78  CI: [-63.72, 91.28] |

SEM = standard error of the mean

md = mean difference

CI = confidence interval

**Bold – Model with highest magnitude accuracy**


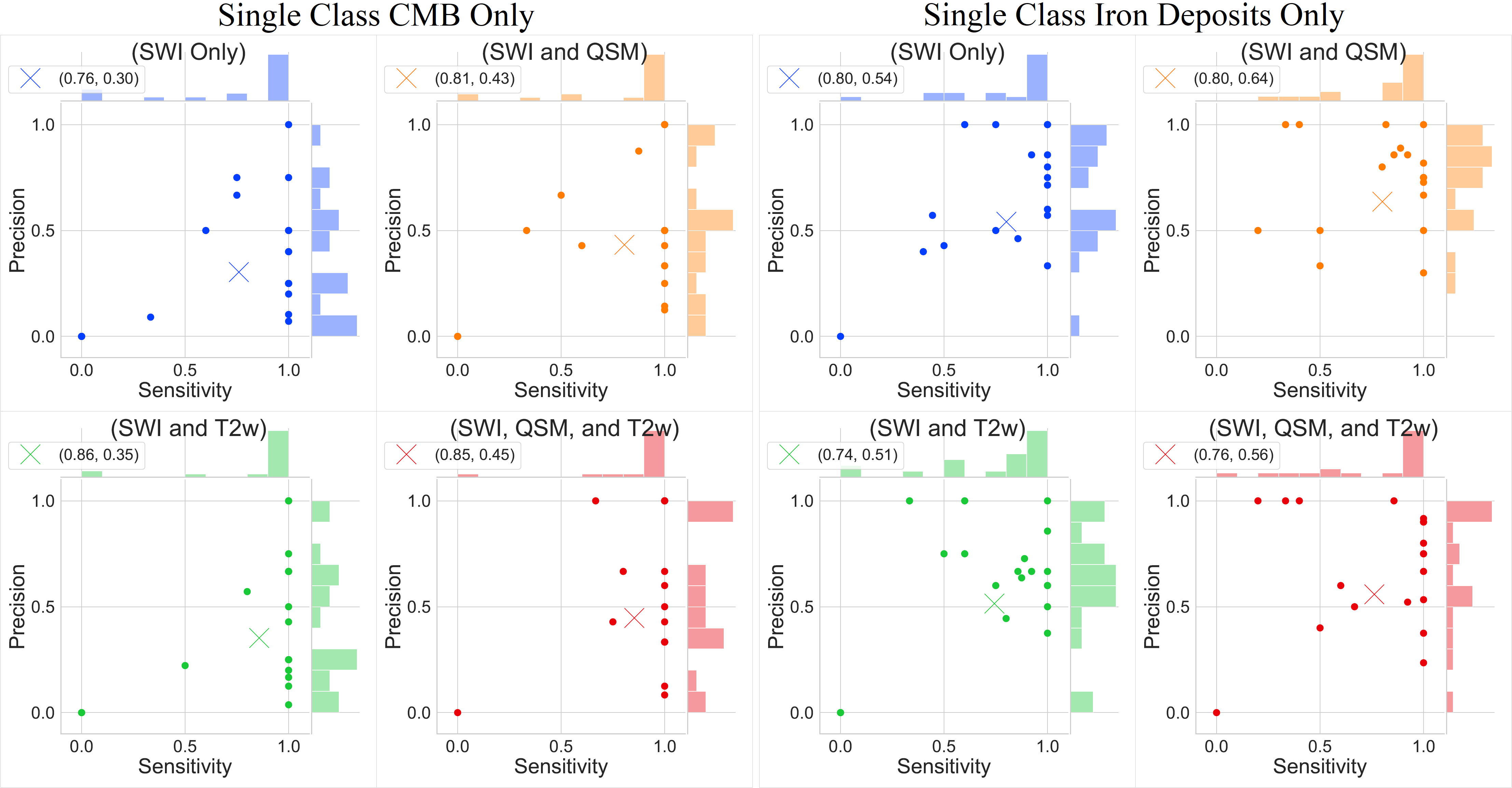


Supplementary Figure 7: Joint scatterplots of the sensitivity vs precision of all single class experiments predicting CMBs and non-hemorrhage iron deposits when excluding the outlier participant. (Left) all CMB only experiments and (Right) all iron deposits only experiments. In each subplot, the round points indicate the individual participants’ sensitivity and precision evaluated with leave-one-out cross-validation, and the X indicates the mean sensitivity and precision. The legend at the upper left corner of each subplot shows the coordinates of X. In each subplot, histograms of the sensitivity and precision are displayed along the upper and right axes.


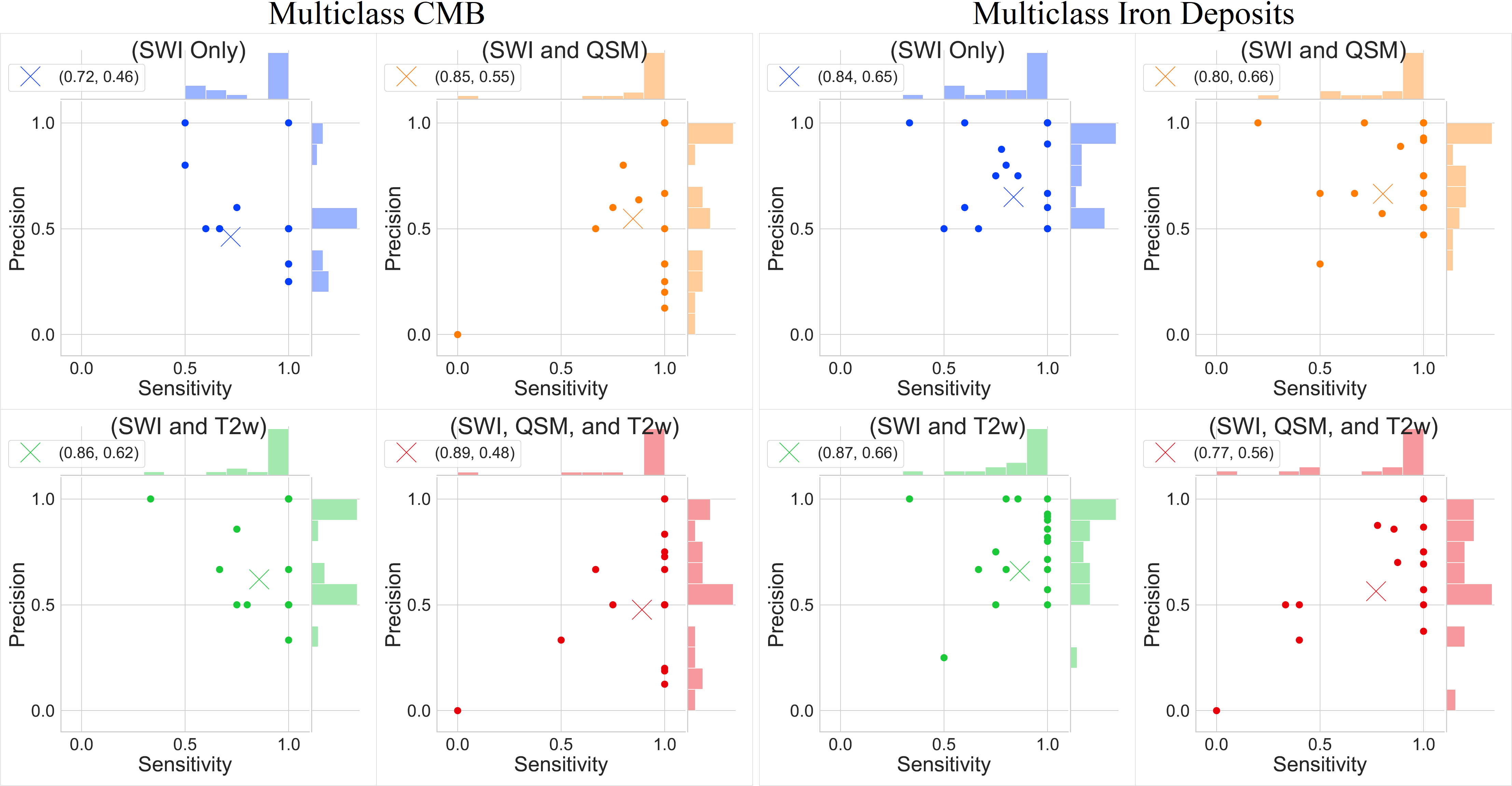


Supplementary Figure 8: Joint scatterplots of the sensitivity vs precision of all multiclass experiments predicting CMBs and non-hemorrhage iron deposits when excluding the outlier. (Left) all evaluations for CMBs and (Right) all evaluations for iron deposits. In each subplot, the round points indicate the individual participants’ sensitivity and precision evaluated with leave-one-out cross-validation, and the X indicates the mean sensitivity and precision. The legend at the upper left corner of each subplot shows the coordinates of X. In each subplot, histograms of the sensitivity and precision are displayed along the upper and right axes.

# Section 4. Additional Experimental Results

## 4.1 Original U-Net

We implemented the original U-Net model as described in reference^9^ and used this model to perform a multiclass cross-validated evaluation of the 24 participants. The main difference between our implementation and the original U-Net is in the size of the input and output images. The original U-Net used input and output image size of 572x572 and 388x388, respectively, whereas our implementation used input and output image size of 412x412 and 228x228, respectively. All other network architecture parameters were kept the same as the original U-Net. The results of these multiclass experiments are reported in Supplementary Table 4.1. We note that for both CMB and iron deposits, the model trained with SWI, QSM and T2w had the best performance in terms of average magnitude accuracy. For the experiment using SWI, QSM and T2 for detecting CMBs, we note that the correlation coefficient is negative. This is because the model failed to produce any CMB predictions for the outlier subject.

Supplementary Table 4.1: Experimental result for the number of predicted CMB and iron deposit lesions evaluated against the reference annotation using the original U-Net^9^.

|  | **Experiments** | **Avg Sensitivity ± SEM [CI: lower, upper]** | **Avg Precision ± SEM [CI: lower, upper]** | **Avg Magnitude Accuracy ± SEM [CI: lower, upper]** | **Pearson Correlation Coeff**  **(p-value)** | **Bland-Altman Plot (md, [lower, upper])** |
| --- | --- | --- | --- | --- | --- | --- |
| **Multiclass CMB** | SWI | 0.79 ± 0.07 [0.65, 0.93] | 0.51 ± 0.07 [0.38, 0.65] | 1.08 ± 0.03 [1.03, 1.13] | 0.97  (p=0.000) | md=0.08  CI: [-20.49, 20.65] |
|  | SWI and QSM | 0.83 ± 0.07 [0.7, 0.97] | 0.48 ± 0.07 [0.35, 0.6] | 0.99 ± 0.08 [0.83, 1.15] | 0.98  (p=0.000) | md=0.33  CI: [-20.89, 21.55] |
|  | SWI and T2w | 0.80 ± 0.07 [0.66, 0.95] | 0.49 ± 0.08 [0.34, 0.64] | 1.1 ± 0.05 [1, 1.2] | 0.83  (p=0.000) | md=0.38  CI: [-32.62, 33.37] |
|  | **SWI, QSM and T2w** | **0.82 ± 0.07 [0.68, 0.96]** | **0.52 ± 0.07 [0.38, 0.66]** | **1.11 ± 0.04 [1.03, 1.2]** | **-0.21**  (p=0.326) | **md=3.21**  **CI: [-44.91, 51.33]** |
|  |  |  |  |  |  |  |
| **Multiclass Iron Deposits** | SWI | 0.74 ± 0.08 [0.58, 0.89] | 0.62 ± 0.09 [0.45, 0.79] | 1.1 ± 0.1 [0.91, 1.29] | 0.83  (p=0.000) | md=12.33  CI: [-100.56, 125.22] |
|  | SWI and QSM | 0.78 ± 0.07 [0.64, 0.92] | 0.59 ± 0.08 [0.44, 0.74] | 1.11 ± 0.07 [0.98, 1.25] | 0.93  (p=0.000) | md=16.62  CI: [-56.09, 89.34] |
|  | SWI and T2w | 0.80 ± 0.07 [0.67, 0.93] | 0.58 ± 0.08 [0.43, 0.73] | 1.09 ± 0.07 [0.95, 1.23] | 0.86  (p=0.000) | md=14.83  CI: [-79.38, 109.05] |
|  | **SWI, QSM and T2w** | **0.82 ± 0.05 [0.72, 0.92]** | **0.61 ± 0.07 [0.47, 0.76]** | **1.14 ± 0.04 [1.06, 1.22]** | **0.84**  (p=0.000) | **md=20.42**  **CI: [-83.54, 124.37]** |

SEM = standard error of the mean

md = mean difference

CI = confidence interval

**Bold – Model with highest magnitude accuracy**

## 4.2 DEEPMIR with Reduced Layers

For this experiment, we modified the proposed multiclass DEEPMIR architecture to have 4 encoding (downsampling) and decoding (up-sampling) layers connected by central convolution block, thereby having 1 less layer than the model described in the main manuscript. All other training/testing parameters remained the same as the experiments in the main manuscript. The results of this experiment are reported in Supplementary Table 4.2.

For CMBs, the standard DEEPMIR model (in the main manuscript) has overall better average sensitivity for all four experiments, but the average precision is worse for two of the experiments. The standard DEEPMIR experiments have smaller mean differences and the Bland-Altman confidence intervals are within similar ranges (except for the experiment with SWI, QSM and T2w). We note that for the standard DEEPMIR model, the best performing experiment (in terms of average magnitude accuracy) was with SWI and QSM (1.15 ± 0.07) and for the modified architecture the best performance was seen in the experiment with SWI, QSM and T2w (1.13 ± 0.08).

For iron deposits, the modified architecture has a slightly improved average sensitivity, but the average precision is slightly worse. The mean difference is smaller, and the Bland-Altman confidence intervals have similar ranges (except for the experiment with SWI and QSM). For both sets of experiments, we note that the experiment with SWI and QSM is the best performing model in terms of average magnitude accuracy.

Supplementary Table 4.2.: Experimental result for the number of predicted CMB and iron deposit lesions evaluated against the reference annotation using the modified DEEPMIR model.

|  | **Experiments** | **Avg Sensitivity ± SEM [CI: lower, upper]** | **Avg Precision ± SEM [CI: lower, upper]** | **Avg Magnitude Accuracy ± SEM [CI: lower, upper]** | **Person Correlation Coeff**  **(p-value)** | **Bland-Altman Plot (md, [lower, upper])** |
| --- | --- | --- | --- | --- | --- | --- |
| **Multiclass CMB** | SWI | 0.74 ± 0.09  [0.56, 0.91] | 0.38 ± 0.07  [0.25, 0.51] | 0.96 ± 0.09  [0.79, 1.13] | 0.96  (p=0.000) | md=0.96  CI: [-26.97, 28.89] |
|  | SWI and QSM | 0.78 ± 0.08  [0.63, 0.93] | 0.48 ± 0.07  [0.35, 0.61] | 1.07 ± 0.04  [0.99, 1.15] | 0.99  (p=0.000) | md=0.92  CI: [-19.80, 21.64] |
|  | SWI and T2w | 0.76 ± 0.07  [0.62, 0.9] | 0.55 ± 0.08  [0.4, 0.71] | 1.11 ± 0.04  [1.03, 1.19] | 0.97  (p=0.000) | md=2.08  CI: [-29.61, 33.77] |
|  | **SWI, QSM and T2w** | **0.78 ± 0.08**  **[0.62, 0.94]** | **0.58 ± 0.08**  **[0.42, 0.75]** | **1.13 ± 0.08**  **[0.97, 1.29]** | **0.99**  **(p=0.000)** | **md=1.22**  **CI: [-16.55, 18.98]** |
|  |  |  |  |  |  |  |
| **Multiclass Iron Deposits** | SWI | 0.79 ± 0.07  [0.65, 0.92] | 0.66 ± 0.08  [0.51, 0.82] | 1.14 ± 0.07  [1, 1.29] | 0.92  (p=0.000) | md=11.92  CI: [-58.30, 82.13] |
|  | **SWI and QSM** | **0.81 ± 0.07**  **[0.67, 0.95]** | **0.73 ± 0.07**  **[0.59, 0.87]** | **1.24 ± 0.03**  **[1.18, 1.29]** | **0.75**  **(p=0.000)** | **md=8.17**  **CI: [-136.90, 153.23]** |
|  | SWI and T2w | 0.77 ± 0.07  [0.64, 0.9] | 0.58 ± 0.08  [0.43, 0.74] | 1.13 ± 0.05  [1.03, 1.23] | 0.88  (p=0.000) | md=13.88  CI: [-74.69, 102.44] |
|  | SWI, QSM and T2w | 0.91 ± 0.03  [0.86, 0.97] | 0.62 ± 0.07  [0.48, 0.76] | 1.19 ± 0.03  [1.13, 1.24] | 0.93  (p=0.000) | md=12.48  CI: [-56.33, 81.29] |

SEM = standard error of the mean

md = mean difference

CI = confidence interval

**Bold – Model with highest magnitude accuracy**

# Section 5. Additional Figures


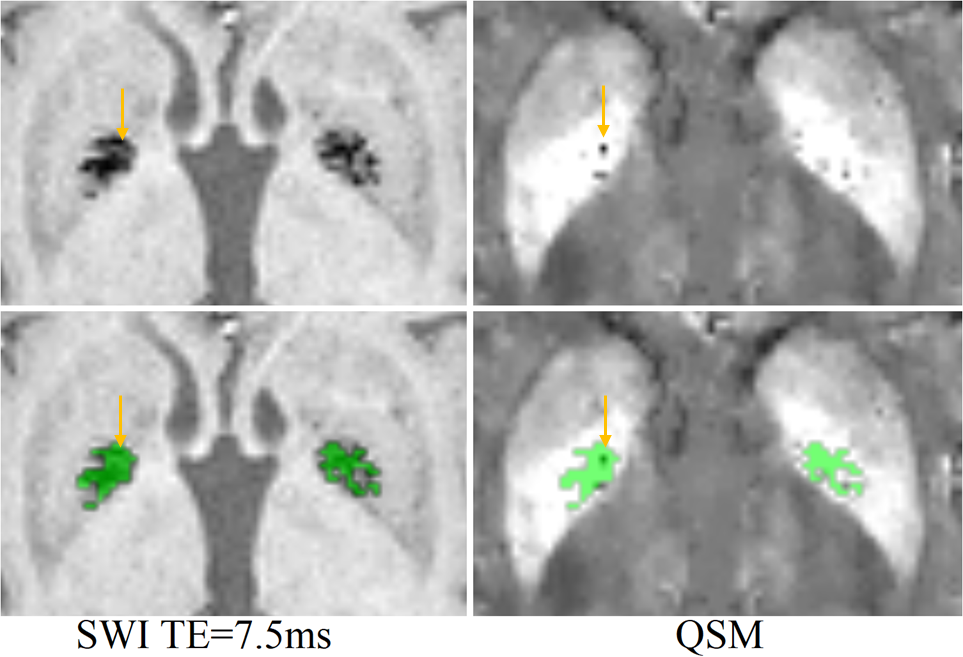


Supplementary Figure 9: Prediction of non-hemorrhage iron deposits by a multiclass standard DEEPMIR model trained with SWI only. (Top row) Mineralization, indicated by the yellow arrow is present in the basal ganglia, and clearly visible in the corresponding QSM. (Bottom row) Incorrect prediction of the mineralization (in translucent green).


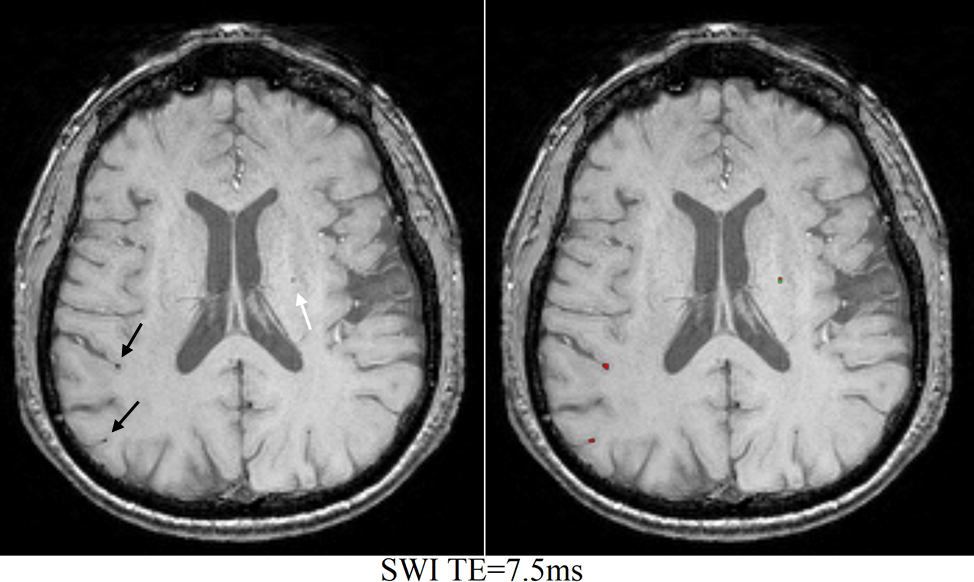


Supplementary Figure 10: Examples of incorrect predictions of CMBs using the standard multiclass DEEPMIR model trained with SWI only. (Left) Microbleed mimics such as sulci (black arrows) and veins (white arrow). (Right) The incorrect predictions of the sulci and vein.


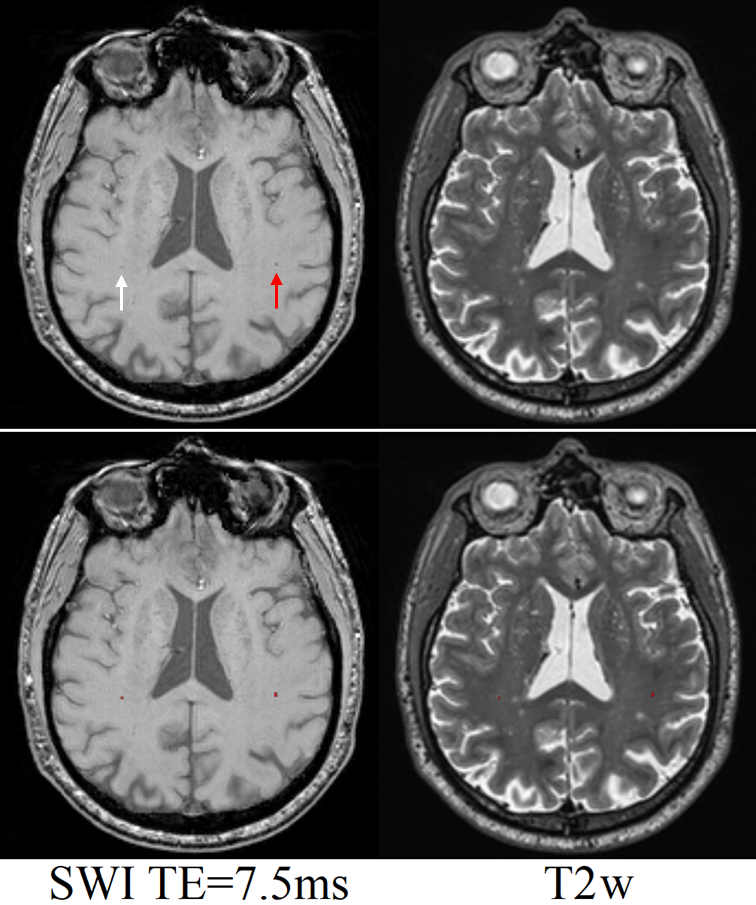


Supplementary Figure 11. An example of an incorrect prediction of CMBs (in red) from the standard multiclass DEEPMIR model trained with SWI and T2w. (Top row) The white arrow shows a vein while the red arrow shows a true CMB. (Bottom row) The incorrect and correct segmentation of the vein and CMB, respectively by the model.


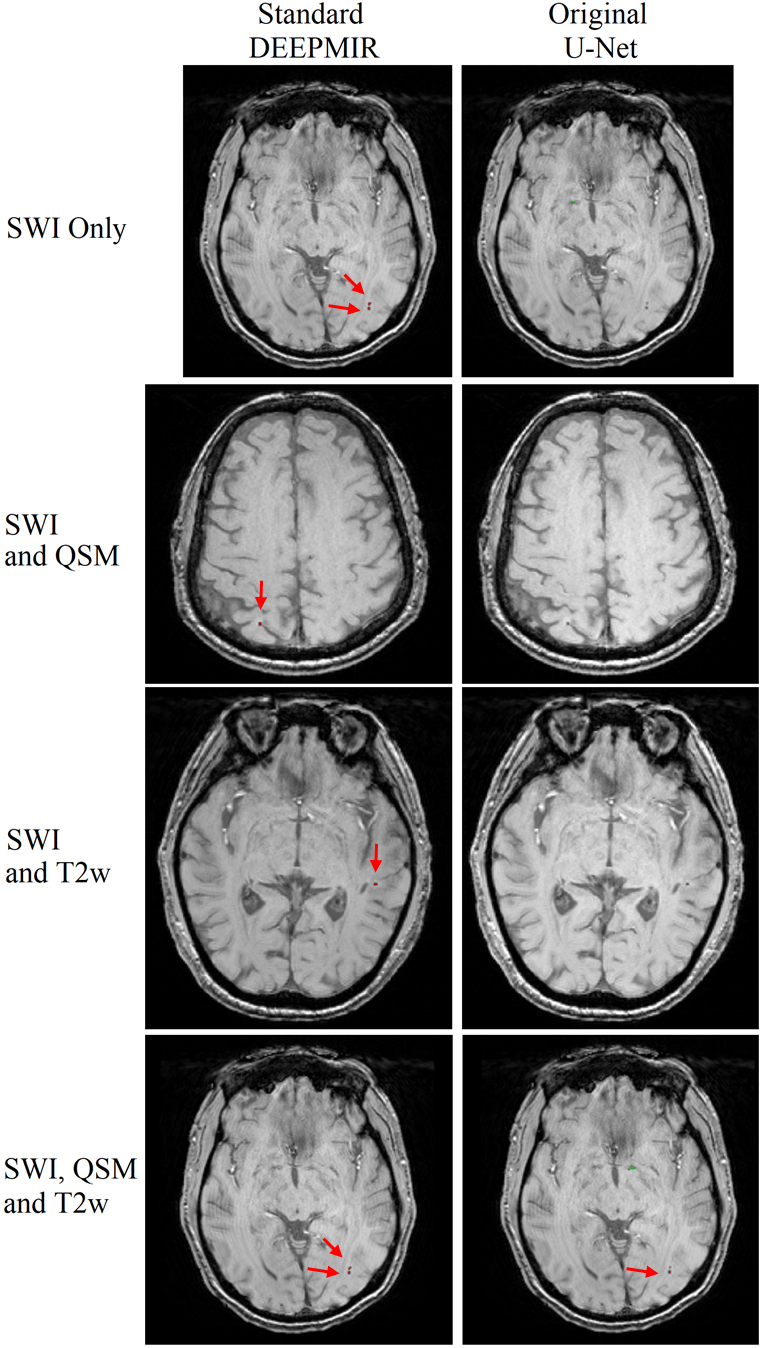


Supplementary Figure 12. A comparison of CMB predictions (red arrows) between the standard DEEPMIR model (left column) and the original U-Net model (right column) for experiments with all the combinations of imaging modalities. These are examples of the original U-Net failing to segment small CMBs.


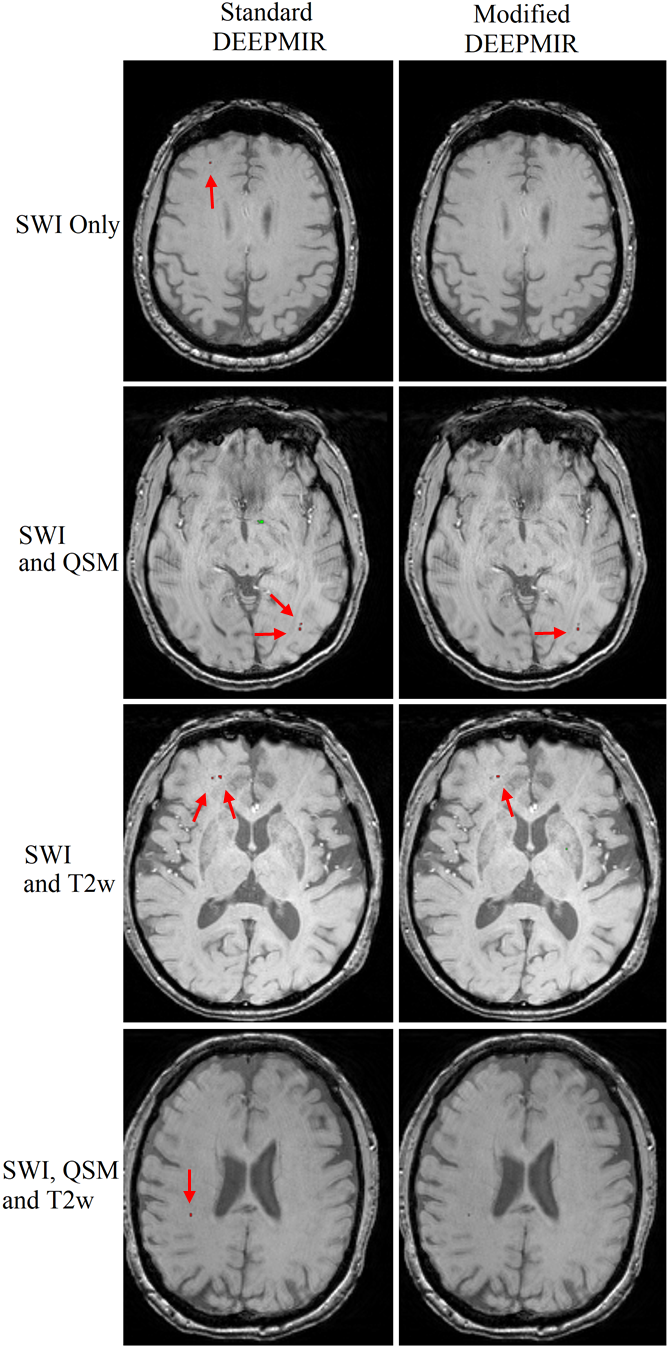


Supplementary Figure 13. A comparison of CMB predictions (red arrows) between the standard DEEPMIR model (left column) and the modified DEEPMIR model with 1 less layer (right column). These are examples of the modified DEEPMIR model failing to segment small CMBs.

# References

1 Ayaz, M., Boikov, A. S., Haacke, E. M., Kido, D. K. & Kirsch, W. M. Imaging cerebral microbleeds using susceptibility weighted imaging: one step toward detecting vascular dementia. *Journal of Magnetic Resonance Imaging* **31**, 142-148 (2010).

2 Li, N., Wang, W. T., Sati, P., Pham, D. L. & Butman, J. A. Quantitative assessment of susceptibility‐weighted imaging processing methods. *Journal of Magnetic Resonance Imaging* **40**, 1463-1473 (2014).

3 Li, N., Wang, W.-T., Sati, P., Pham, D. L. & Butman, J. A. in *Medical Imaging 2012: Biomedical Applications in Molecular, Structural, and Functional Imaging.* 83171S (International Society for Optics and Photonics).

4 Gregoire, S. *et al.* The Microbleed Anatomical Rating Scale (MARS): reliability of a tool to map brain microbleeds. *Neurology* **73**, 1759-1766 (2009).

5 Cordonnier, C. *et al.* Improving interrater agreement about brain microbleeds: development of the Brain Observer MicroBleed Scale (BOMBS). *Stroke* **40**, 94-99 (2009).

6 Vernooij, M. *et al.* Prevalence and risk factors of cerebral microbleeds: the Rotterdam Scan Study. *Neurology* **70**, 1208-1214 (2008).

7 Lee, S.-H., Kwon, S.-J., Kim, K. S., Yoon, B.-W. & Roh, J.-K. Cerebral microbleeds in patients with hypertensive stroke. *Journal of neurology* **251**, 1183-1189 (2004).

8 Wilcoxon, F. in *Breakthroughs in statistics* 196-202 (Springer, 1992).

9 Ronneberger, O., Fischer, P. & Brox, T. in *International Conference on Medical image computing and computer-assisted intervention.* 234-241 (Springer).
